# Endothelial Glycocalyx Degradation in Critical Illness and Injury

**DOI:** 10.3389/fmed.2022.898592

**Published:** 2022-07-08

**Authors:** Eric K. Patterson, Gediminas Cepinskas, Douglas D. Fraser

**Affiliations:** ^1^Centre for Critical Illness Research, Lawson Health Research Institute, London, ON, Canada; ^2^Department of Medical Biophysics, Western University, London, ON, Canada; ^3^Department of Pediatrics, Western University, London, ON, Canada; ^4^Department of Physiology and Pharmacology, Western University, London, ON, Canada; ^5^Department of Clinical Neurological Sciences, Western University, London, ON, Canada; ^6^Children’s Health Research Institute, Lawson Health Research Institute, London, ON, Canada

**Keywords:** glycocalyx, inflammation, sepsis, trauma, endothelium

## Abstract

The endothelial glycocalyx is a gel-like layer on the luminal side of blood vessels that is composed of glycosaminoglycans and the proteins that tether them to the plasma membrane. Interest in its properties and function has grown, particularly in the last decade, as its importance to endothelial barrier function has come to light. Endothelial glycocalyx studies have revealed that many critical illnesses result in its degradation or removal, contributing to endothelial dysfunction and barrier break-down. Loss of the endothelial glycocalyx facilitates the direct access of immune cells and deleterious agents (e.g., proteases and reactive oxygen species) to the endothelium, that can then further endothelial cell injury and dysfunction leading to complications such as edema, and thrombosis. Here, we briefly describe the endothelial glycocalyx and the primary components thought to be directly responsible for its degradation. We review recent literature relevant to glycocalyx damage in several critical illnesses (sepsis, COVID-19, trauma and diabetes) that share inflammation as a common denominator with actions by several common agents (hyaluronidases, proteases, reactive oxygen species, etc.). Finally, we briefly cover strategies and therapies that show promise in protecting or helping to rebuild the endothelial glycocalyx such as steroids, protease inhibitors, anticoagulants and resuscitation strategies.

## Glycocalyx Overview

The glycocalyx is a complex, negatively charged, gel-like layer on the luminal side of endothelial cells (ECs), which is composed of glycosaminoglycans (GAGs) that are bound to membrane-spanning proteins that anchor the structure. It is a dynamic structure, with its various components consistently shed and replaced; in particular, hyaluronic acid (HA) is turned over rapidly (1). The structure of the glycocalyx depends on the organ and type of endothelium, for example: the endothelium can be continuous, fenestrated or sinusoid (2); the thickness of the glycocalyx varies with blood vessel size, ranging from approximately 0.5 μm thick in capillaries (3) to 2.5 μm in arteries (4); and the pulmonary endothelial glycocalyx can be more than 2 times thicker than in muscle (5).

**FIGURE 1:**
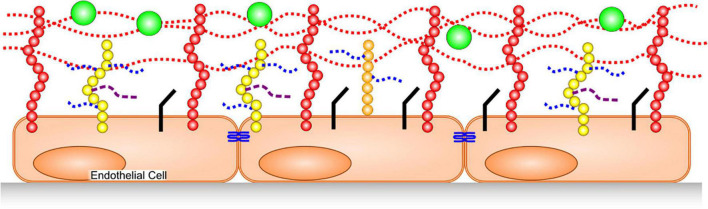
Glycocalyx Structure. HA, dotted red lines, is bound to CD44 in membranes (red). HS (dotted blue) and CS (dashed purple) are covalently bound to syndecans (yellow) and glypican-1 (orange). Junctional proteins (blue), cellular adhesion molecules (black) adsorbed plasma proteins (green).

The GAGs that constitute the glycocalyx are primarily heparan sulfate (HS), chondroitin sulfate (CS) and hyaluronic acid (also called hyaluronan or hyaluron). HS and CS are sulfated, and covalently bound to trans-membrane syndecans-1,4 or membrane-anchored glypican-1. HA is not sulfated, and is a much larger polymer than the former two, with a molecular weight often in the hundreds of kilodaltons to megadalton range. Additionally, hyaluronic acid is non-covalently bound to multiple membrane-spanning CD44 molecules that are cell-surface glycoproteins involved in cell–cell interactions, cell adhesion, and migration (6) (Figure 1).

The glycocalyx has a net negative charge that helps determine interactions with proteins. In particular, it can adsorb positively charged regions of some plasma proteins and compliments endothelial barrier function by acting as the first barrier to plasma protein (which are mostly negatively charged, e.g., albumin) leakage into the interstitium. Further, by preventing protein leak from the vasculature, the glycocalyx helps to maintain osmotic pressure toward the blood vessel’s lumen, thereby inhibiting water passage into tissues. Finally, the glycocalyx is anti-thrombotic/profibrinolytic, as well as anti-neutrophil/leukocyte attachment. These latter mechanisms are achieved through HS binding of positively charged regions on antithrombin (7, 8) and by burying cellular adhesion molecules within the depth of the intact glycocalyx (9). HS in the glycocalyx not only binds antithrombin, but also enhances its inhibition of thrombin and factors IXa, Xa (7) and XIa (10).

**FIGURE 2:**
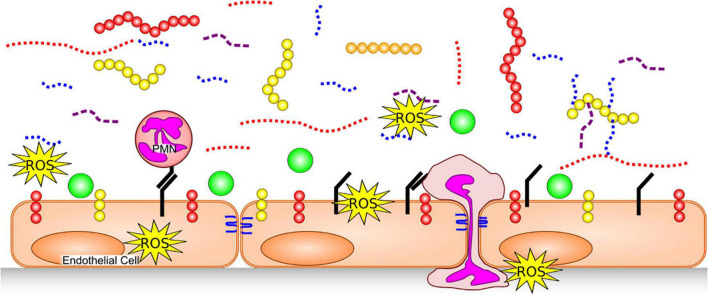
Glycocalyx Degradation. The endothelial glycocalyx is degraded by proteases [which remove core proteins’ ectodomains: syndecans (yellow), glipicans (orange) and CD44 (red)], GAG-degrading enzymes and ROS, leaving free fragments of HA (red dotted), HS (blue dotted) and CS (purple dashed). This exposes cellular adhesion molecules (black) on the endothelial surface, allowing easier binding of white blood cells [e.g., neutrophils (PMNs)] and platelets. Neutrophils can release additional proteases, further damaging core glycocalyx proteins, junctional proteins (blue) and produce additional ROS. With a degraded glycocalyx, blood flows closer to the endothelial cells and plasma proteins (green), can access the endothelial surface.

Exposure to inflammatory stimuli, including TNF-α (11) and endotoxin (12), as well as inflammatory states such as sepsis (13, 14), degrade the glycocalyx by triggering release or activity of various enzymes and/or reactive oxygen species (ROS) (Figure 2). Glycocalyx degradation exposes the underlying cell adhesion molecules, thereby promoting leukocyte (15) and platelet (16) adhesion and inducing a pro-thrombotic state. The barrier function of the endothelium is compromised by glycocalyx degradation, increasing its permeability to fluids and macromolecules, as well as leukocyte migration. Additionally, denuding the glycocalyx exacerbates EC exposure to proteases capable of degrading GAG-anchoring proteins, or cell junctions. A non-exhaustive list of agents which are thought to degrade various components of the endothelial glycocalyx in disease is provided in Table 1.

**TABLE 1:** Glycocalyx-degrading agents in critical illness.

Component	Degrading agent during critical illness	References
Hyaluronic acid	HYAL1, HYAL2, TMEM2, ROS	(17, 20–24)
Heparan sulfate	HYAL1, HEPase1, CTAP-III	(25–27, 29)
Chondroitin sulfate	HYAL4	(30)
Syndecan-1	MMP2,3,7,9, and 14, ADAM17	(35, 37, 38)
Syndecan-4	MMP2,3,7,9, and 14, ADAM17	(37, 38)
CD44	ADAM15, MMP14	(36, 126)

*ADAM, a disintegrin and metalloproteinase; CTAP-III, connective tissue activating peptide-III; HEPase1, Heparanase-1; HYAL, hyaluronidase; MMP, matrix metalloproteinase; TMEM2, transmembrane 2; ROS, reactive oxygen species.*

### Hyaluronic Acid

The inflammatory mechanism(s) and enzyme(s) that are responsible for HA degradation in the vasculature are somewhat controversial. Hyaluronidase-1 was characterized from serum nearly 30 years ago (17), but its pH optimum of approximately 3.5 (17) suggests that it would be primarily active in lysosomes and would have limited activity in plasma. Indeed, data refutes hyaluronidase-1 as a sheddase in sepsis (18). Hyaluronidase-2 appears to have a membrane-tethered form (19), but again its pH optimum suggests limited activity in plasma, though platelet surface-bound hyaluronidase-2 degrades high molecular weight HA on the endothelial surface into pro-inflammatory fragments under neutral pH *in vitro* (20). Further, when hyaluronidase-2 and CD44 are co-expressed on HEK293 cells they extracellularly degrade HA with a pH optimum between 6.0 and 7.0 (21). The relatively low pH of inflamed and/or poorly perfused tissues could impart significant activity to these enzymes, especially hyaluronidase-2 since endothelial cells co-express CD44. More recently the protein transmembrane 2 (TMEM2) was shown to have significant hyaluronidase activity (22). TMEM2 is highly expressed in endothelial cells, at least of dermal, lymph or liver origin (22, 23) and functions to degrade free plasma HA in the liver (23). At present, it is unclear if TMEM2 instigates HA shedding or degradation during inflammation in non-liver blood vessels.

Changes in cell-surface HA during inflammation is not limited to degradation only. HA can form cable-like structures on the endothelial surface that serve as an attachment site for monocytes and platelets regardless of their inflammatory status (19). In addition to enzymatic cleavage, ROS such as hydroxyl radicals, hypochlorous acid, and peroxynitrite directly degrade HA (19, 24).

### Heparan Sulfate

Heparanase-1 is expressed in platelets (25) and neutrophils (26), and it is located extracellularly as well as in lysosomes (27). It degrades HS at sites of inflammation or injury, thereby contributing to leukocyte attraction (27). Heparanase-1 activity is optimal at the acidic pH of approximately 6.4, and is somewhat active below pH 6.8 (28), suggesting it has activity in the relatively low pH environment of inflammation. Additionally, heparan sulfate is cleaved by connective tissue activating peptide-III (CTAP-III), an N-truncated version of CXCL7 with endoglycocidic heparinase activity (29). CTAP-III is expressed in both platelets and neutrophils at similar levels to heparanase-1 (27, 29). Despite the optimal pH of 5.8, CTAP-III has significant activity up to approximately pH 7.0 (29) suggesting it too has activity in inflammatory environments.

### Chondroitin Sulfate

There are 3 known human hydrolases capable of degrading CS outside of lysosomes; SPAM1 (also called PH-20, located mostly in the sperm acrosome), hyaluronidase-1 (see above) and hyaluronidase-4 (30). Hyaluronidase-4, (also called CS hydrolase) has a membrane-bound form present in neutrophils that preferentially hydrolyze CS over HA (30).

### Glycosaminoglycan-Related Effects

In addition to the direct mechanical and spatial effects, removing HA from the endothelial surface has other pro-inflammatory and pro-thrombotic effects, such as removing proteins bound or adsorbed to the glycocalyx. HA binds protease inhibitors such as the inter-alpha-trypsin inhibitors, and tumor necrosis factor-stimulated gene 6, which reduce neutrophil adhesion, ROS generation, hyaluronidase activity, complement activation and contributes to matrix metalloproteinase (MMP) inhibition (19, 31). Additionally, degraded HA fragments (< 500 kDa) appear to have pro-inflammatory properties of their own (19). Tissue factor pathway inhibitor (TFPI) binds heparan sulfate molecules in the glycocalyx (32), helping it to maintain close proximity to the endothelium; as HS is removed, the pro and anti-thrombotic balance tilts toward complement activation and thrombus formation. Additionally, hyaluronidase treatment of primary human pulmonary vascular endothelial cells *in vitro* results in decreased NO production (33), which is necessary for platelet adhesion to the endothelial surface.

### Glycosaminoglycan-Anchoring Proteins

Glycocalyx compromise is not only due to GAG degradation, but also *via* cleavage of the proteins that anchor the GAGs to the endothelial plasma membranes. Pre-treating tissues with the broad-spectrum MMP inhibitor doxycycline inhibited glycocalyx shedding and leukocyte adhesion in a murine inflammation model (34), demonstrating the crucial role for MMP-induced glycocalyx breakdown. More specifically, the membrane-bound MMP14 has been shown to cleave syndecan-1 (35) and CD44 (36). Additionally, syndecan-1 and syndecan-4 are cleaved by MMPs 2, 3, 7, 9, and 14, (37) as well as ADAM17 (38).

## Disease-Specific Glycocalyx Alterations

Several major glycocalyx components have been used as biomarkers to distinguish sub-populations in critical illness and injury, as well as to prognosticate outcomes. A list of the literature cited herein, with the component studied, biochemical thresholds and/or statistical value is provided in Table 2.

**TABLE 2:** Glycocalyx components used as biomarkers.

Component	Threshold/ Value	Population, measure	References
PBR	AUC = 0.778	Sepsis, in-hospital mortality	(42)
	AUC = 0.75	COVID-19, 60-day mortality	(56)
Syndecan-1	OR = 1.850	Sepsis, in-hospital mortality	(14)
	AUC = 0.781	Sepsis, in-hospital mortality	(42)
	898 ng/mL, AUC = 0.801	Sepsis, 90-day mortality	(44)
	HR = 1.95	Septic shock, 90-day mortality	(46)
	813.8 ng/mL, AUC = 0.783	COVID-19, in-hospital mortality	(61)
	*p* < 0.0001	COVID-19, critical vs severe	(63)
	OR = 1.01, p = 0.043	Trauma, 30-day mortality	(74)
	40 ng/mL, AUC = 0.71, OR = 2.23	Trauma, 30-day mortality	(76)
	*p* < 0.05	T1D, nephropathy vs not	(95)
	*p* < 0.0001	T1D, microalbuminuria vs not	(96)
Hyaluronic acid	p = 0.006	Sepsis, 90-day mortality	(43)
	441 ng/mL, AUC = 0.827	Sepsis, 90-day mortality	(44)
	*p* < 0.001	COVID-19, ICU vs non-ICU	(60)
	*p* < 0.05	Trauma, coagulopathy vs not	(77)
	*p* < 0.05	T1D, microalbuminuria vs not	(90)
Heparan sulfate	p = 0.02	Sepsis, 90-day mortality	(43)
	*p* < 0.05	Sepsis, 28-day mortality	(45)
	*p* < 0.05	COVID-19, ICU vs non-ICU	(60)
	*p* < 0.001	COVID-19, critical vs moderate	(63)
	*p* < 0.05	Trauma, autoheparinization vs not	(71)

*AUC, area under the curve for a receiver-operator characteristic; HR, hazard ratio; ICU, intensive care unit; OR, odds ratio; PBR, perfused boundary region, a measure of glycocalyx thickness; T1D, type-1 diabetes.*

### Sepsis

Sepsis is defined as life-threatening organ dysfunction caused by a dysregulated host response to infection (39). Degradation of the glycocalyx encountered during sepsis conforms to the pattern seen in many severe inflammatory diseases. The systemic pro-inflammatory stimuli (e.g., cytokines, lipopolysaccharide, ROS) produced during sepsis are the initiating factors in a feed-forward cascade of inflammation and glycocalyx degradation (40, 41). The degraded glycocalyx allows the binding and extravasation of leukocytes, as well as platelet recruitment (40, 41), thereby resulting in further inflammation and greater risk of thrombosis. Further, glycocalyx loss leads to capillary leaking that contributes to systemic edema, hypovolemia, and along with thrombi, contribute to circulatory dysfunction (41). Ultimately, the above events result in hypoperfusion of tissues and organ damage that is a hallmark of sepsis (40, 41).

Measurements of the perfused boundary region in sublingual micro-vessels (a measurement of glycocalyx thickness), showed the glycocalyx is thinner in sepsis non-survivors than survivors, and admission perfused boundary region was associated with hospital mortality (AUC 0.778) (42).

On the day of intensive care unit (ICU) admission, sepsis patients showed a significantly higher median plasma concentration of HA and HS compared to controls, with those who died in the next 90 days displaying a significantly higher concentration within the sepsis population (43). In the same study, plasma HA and HS correlated with IL-6, IL-10, and sequential organ failure assessment (SOFA) score. A later study confirmed higher concentrations of HA, as well as syndecan-1, in sepsis patients at multiple time points of illness (44). HA and syndecan-1 concentrations were also higher for the first five ICU days in severe sepsis (sepsis with acute organ dysfunction) and septic shock (sepsis with refractory hypotension despite adequate fluid loading) patients, when compared to sepsis. Furthermore, HA and syndecan-1 were elevated for at least the first 3 days in septic shock vs. severe sepsis patients. More importantly, in survivors, the HA and syndecan-1 concentrations tended to decrease over the ICU stay, while they tended to slightly increase or stay the same in non-survivors. Both glycocalyx components were correlated with Acute Physiologic Assessment and Chronic Health Evaluation II (APACHE II) and SOFA scores; cutoff values of < 441 ng/mL HA on ICU day 7 or < 898 ng/mL syndecan-1 on ICU day 5 were found to predict 90 and 87% survival, respectively (44). Another study showed that plasma HA is increased in septic shock patients compared to healthy controls, but not pancreatitis patients (18). Interestingly, this same study showed that plasma hyaluronidase activity was actually lower in septic shock patients, compared to healthy controls. While plasma hyaluronidase measurements may prove to be interesting diagnostically or functionally, it’s not clear if the plasma hyaluronidase (HYAL1), can act as a HA sheddase in either sepsis or other inflammatory diseases. HYAL2 is membrane-bound and has a pH optimum closer to that of inflamed tissues, though its activity would not be present in plasma tests due to its anchoring in the plasma membrane.

Patients with non-pulmonary sepsis, on mechanical ventilation and with an APACHE II ≥ 25, had higher plasma syndecan-1 concentrations on day 2 of ICU admission with levels that were significantly correlated with acute respiratory distress syndrome (ARDS), though this was not true of pulmonary sepsis patients (pneumonia or aspiration of gastric contents) (14). In those patients that developed ARDS, syndecan-1 levels were higher than in those that developed ARDS from non-pulmonary sepsis. Additionally, syndecan-1 levels were associated with vasopressor requirements, and circulatory, hepatic, renal, and coagulation failures (14). Moreover, higher syndecan-1 concentrations were independently predictive of in-hospital mortality. There was no correlation between syndecan-1 and plasma myeloperoxidase concentrations, used as a marker of neutrophil activation (a source of proteases).

While syndecan-1, HA and HS were significantly higher in septic shock patients compared to sepsis patients, and syndecan-1 was good at predicting progression to septic shock (81.8% sensitivity, 78.3% specificity), interestingly, it was not higher in non-survivors, vs. survivors (45). In contrast, the authors did identify significantly increased plasma concentrations of HS in non-survivors, compared to survivors (204.5 vs. 158.9 ng/mL). All three glycocalyx components measured had a weak to moderate correlation with disease severity as assessed by either APACHE II or SOFA scores (*r* ≤ 0.5) (45). Further, plasma syndecan-1 and HA concentrations were found to be significantly higher in a subset of patients with disseminated intravascular coagulation (DIC), and may be useful predictors of DIC, underscoring the importance of the anti-fibrinolytic actions attributed to the glycocalyx.

More recently, syndecan-1 plasma concentrations in septic shock patients were found to be more than double that of healthy volunteers on day 1 of ICU admission and were significantly associated with the general SOFA score and the coagulation SOFA subscale (46). Syndecan-1 concentrations were significantly associated with the need for renal replacement therapy, or slow dialysis provided to hemodynamically unstable patients, as well as the incidence of coagulation failure and 90-day mortality (46). Interestingly, many of these associations were found oppositely correlated with sphingosine 1-phosphate which protects syndecan-1 from shedding.

In sepsis cases, the coagulation system can become pathologically activated, resulting in DIC and thrombosis. Whole-blood measurements of coagulation in sepsis patients can reveal a hypo-, normal or hyper-coagulable state (47), while traditional lab tests may show the plasma is not hypercoagulable *per se* (48), leading to the hypothesis that endothelial dysfunction may be a major contributing factor to DIC. Indeed, glycocalyx degradation upsets the interplay between blood and the anti-coagulation/anti-thrombotic properties of the glycocalyx. In particular, removing HS will also remove the bound antithrombin from the endothelial surface, resulting in increased fibrin formation, thereby allowing thrombin easier access to membrane-spanning thrombomodulin to activate protein C. HS shedding and proteolysis also leads to decreased HS-bound TFPI in sepsis (49).

Monitoring the progression of glycocalyx damage markers (e.g., HA or syndecan-1) may prove to be useful to assess the progression of sepsis and predict survival. Currently, there is no single biomarker proven to predict sepsis progression or mortality, and considering the complexity of sepsis, a single biomarker is not likely to emerge. So far, the enzyme(s) responsible for HA shedding/degradation from the endothelium have not been positively identified. More work is required to positively identify the precise mechanism of HA shedding, whether it’s enzymatic or chemically induced (e.g., ROS).

### Coronavirus Disease 2019

While Coronavirus disease 2019 (COVID-19) is a newly emerged disease associated with SARS-CoV-2, multiple studies quickly identified microvascular injury and glycocalyx degradation as major pathophysiological disease mechanisms. Similar to bacterial sepsis, COVID-19 glycocalyx damage follows a familiar pattern and the glycocalyx degradation and endothelial damage seen in COVID-19 leads to a pro-thrombotic state (33, 50, 51) that in severe cases results in multi-organ thrombosis (50). Patient thrombosis has a negative effect on patient outcomes (52), with a thrombotic event being independently associated with mortality in COVID-19 patients (53, 54). Therapeutic doses of heparin reduce the likelihood of progression to intubation and death in non-critically ill hospitalized COVID-19 patients (52), highlighting the importance of thrombosis in COVID-19 disease.

Early in the pandemic, we investigated plasma from age- and sex-matched subjects, including COVID-19 ICU patients, non-COVID-19 ICU patients and healthy controls (33). Compared with healthy control subjects, COVID-19 positive patients had higher plasma von Willebrand factor, and glycocalyx-degradation products (chondroitin sulfate and syndecan-1). When compared with COVID-19 negative sepsis patients, COVID-19 positive patients had persistently higher soluble P-selectin, hyaluronic acid, and syndecan-1, particularly on ICU day 3 and thereafter. In fact, syndecan-1 continued to increase over the 7 days that COVID-19 patients were tested. Our data suggested that glycocalyx degradation was greater in COVID-19 patients, as opposed to age- and sex-matched non-COVID-19 ICU patients, perhaps explaining the greater risk of thrombosis in COVID-19 (33). As proof of principle, we removed surface hyaluronic acid from human pulmonary microvascular endothelial cells with hyaluronidase treatment resulting in depressed nitric oxide production, an instigating mechanism for platelet adhesion to the microvascular endothelium.

Additionally, we published a case report of a 15-year old female admitted to hospital with multi-system inflammatory syndrome associated with COVID-19 (MIS-C), and demonstrated that plasma HA was increased almost 7-fold over age and sex-matched healthy controls (55). Further, MMP7, which is known to cleave syndecans-1 and 4 (Table 1), was the most up-regulated analyte tested (over 15-fold) (55). These latter data were consistent with endothelial glycocalyx degradation following SARS-CoV-2 infection, albeit with a MIS-C presentation.

Measuring the perfused boundary region of sub-lingual blood vessels has become a useful bedside indicator of glycocalyx damage. Mechanically ventilated COVID-19 patients have been shown to have a thinner glycocalyx, compared to non-ventilated COVID-19 patients or healthy controls (56). The same study showed that plasma concentrations of HA were significantly higher in both non- and ventilated COVID-19 patients compared to controls, while syndecan-1 was higher in ventilated COVID patients compared to both non-ventilated COVID-19 patients and controls. Additionally syndecan-1 predicted development of moderate-to-severe ARDS (AUC 0.91), and a thinner glycocalyx was associated with 60-day mortality (56).

Subsequent studies have reported increased plasma syndecan-1 (57, 58) and HA (57, 59) in COVID-19 patients compared to healthy controls. One study showed plasma HA, HS, and CS were all increased in COVID-19 patients compared to healthy controls, but lower or similar to sepsis patients; though COVID-19 ICU patients had significantly higher concentrations of HA and HS compared to non-ICU COVID patients (60). Additionally, plasma hyaluronidase activity was higher in the ICU COVID-19 patients vs. the non-ICU patients, and plasma MMP2, MMP9 and cathepsin D activities (enzymes which may cleave GAG-anchoring proteins) were significantly increased in COVID-19 patients vs. healthy controls (60). Interestingly, COVID-19 patient plasma HA was also found to be low molecular weight (pro-inflammatory) and bound with inter-alpha inhibitor protein, suggesting that free HA in COVID-19 plasma is a degradation product of ECs rather than increased HA release. HA and hyaluronidase plasma concentrations showed moderate correlations with SOFA score in COVID-19 patients. Studies utilizing cultured endothelial cells treated with COVID-19 patient plasma *in vitro* showed similar changes in HA, as well as hyaluronidase, MMP2, MMP9, and cathepsin activity (60).

Several studies suggest that blood markers of glycocalyx degradation are correlated with disease severity in COVID-19 patients. Serum syndecan-1 concentrations within the first day of ICU admission were significantly higher in non-surviving COVID-19 patients compared to survivors (61). The optimal cut-off value to distinguish survivors was 813.8 ng/mL, as determined by ROC analysis, and Kaplan-Meier analysis showed significantly worse outcomes in COVID-19 patients over the cut-off. Another study found that plasma heparanase activity and plasma heparan concentration were higher in COVID-19 patients compared to healthy controls, and that heparanase activity was significantly higher in ventilated ICU COVID patients vs. non-ICU COVID patients (62). Plasma syndecan-1 was significantly higher in COVID-19 patients classified as “critical” compared to those in the “severe” category; a difference that persisted for 14 days of ICU admission (63). A further study showed plasma HS concentrations were increased in COVID-19 patients compared to healthy controls (59, 64) and were associated with the severity of disease (64).

Even in convalescent COVID-19 patients who were never hospitalized, a persistent increase in serum syndecan-1 concentrations compared to healthy controls was found at a mean of 88 days post symptom onset, and was not significantly different from currently hospitalized COVID-19 patients (65). Another study found decreased glycocalyx thickness (using sublingual perfused boundary region) 4 months after COVID-19 infection (none required mechanical ventilation), which was similar to untreated hypertensive patients (66). While these studies contained relatively few subjects, they suggest glycocalyx degradation also occurs in mild cases of COVID-19, and elevated degradation products can persist for months after acute illness.

SARS-CoV-2 causes direct endothelial damage *via* ACE2 receptors, and ACE2 activation may be modulated by the health and thickness of the glycocalyx (67). In fact, the SARS-CoV-2 spike protein requires HS to aid ACE2 binding (68), a thick, healthy glycocalyx may act as a physical barrier, extending beyond the ACE2 receptor and preventing the virus from accessing the EC (67). However, by the time COVID-19 patients exhibit viremia, one would expect the disease to be advanced and the glycocalyx to be severely damaged *via* the inflammatory response.

Taken together, the published studies strongly suggest that the glycocalyx clearly undergoes a significant amount of degradation from COVID-19, likely contributing to platelet adhesion and increased risk of thrombosis seen in many cases of COVID-19. Thus, therapies to inhibit platelet adhesion (e.g., administration of nitric oxide *via* inhalation or by a donor) and to protect/restore the glycocalyx (e.g., sulodexide and/or sphingosine-1-phosphate) may be therapeutically indicated.

### Trauma—Fluid Resuscitation

Similar to sepsis and COVID-19, trauma can lead to a hypercoagulative state due to the interplay of inflammation and vascular injury. Trauma-induced coagulopathy may begin with a hypercoagulative state that changes to hypcoagulation, or vice-versa, and can depend on several factors including the extent of trauma, the amount and rate of intravascular fluid administered, and the presence of excess fibrinolysis (69, 70). However, as seen below, trauma is consistently associated with glycocalyx degradation. Interestingly, in a subset of trauma patients, glycocalyx degradation, and the release of heparin-like GAGs, including HS, has been suggested to contribute to hypocoagulation, termed auto-heparinization (71). While trauma is known to increase the plasma concentrations of glycocalyx components such as HA, HS, CS, and syndecan-1 compared to healthy controls (72), it is less well known how the glycocalyx is affected by fluid resuscitation. Previous strategies to quickly resuscitate with large amounts of isotonic fluid may not be as beneficial as conservative strategies (73) and may lead indirectly to extra glycocalyx shedding.

Plasma syndecan-1 concentration in trauma patients upon arrival to the emergency department was an independent predictor of mortality after adjusting for age and injury severity (74). Patients above the median syndecan-1 concentration also showed increased markers of inflammation, endothelial activation/injury and fibrinolysis. Trauma patients with a lower plasma colloidal pressure, secondary to uncontrolled hemorrhage together with saline resuscitation, also had increased plasma HA and syndecan-1 compared to trauma patients with normal colloidal pressure (72). Blood syndecan-1 concentrations were elevated in trauma patients after admission to the emergency department, and those with above average syndecan-1 concentrations, had more indicators of microcirculatory dysfunction (75). While microcirculatory dysfunction improved over time and syndecan-1 concentrations decreased, syndecan-1 remained elevated for 30–50 h compared to healthy controls (75).

Trauma patients in the highest quartile of plasma syndecan-1 concentration upon admission to the emergency department had the highest rates of blood transfusion and 30-day in-hospital mortality (76). In fact, a blood concentration of 40 ng/mL syndecan-1 maximized sensitivity and specificity in predicting 24-h in-hospital mortality. Further, patients with plasma syndecan-1 ≥ 40 ng/mL were significantly more injured and had lower median systolic blood pressures and platelet counts (76). Higher plasma syndecan-1 levels translated into poorer outcomes, needing 4 times more blood products, having fewer hospital ventilator-free days and greater mortality.

Syndecan-1 seems to be the most well-studied glycocalyx degradation marker in trauma, however, a small study showed plasma HA concentrations are also significantly associated with acute traumatic coagulopathy and markers of coagulopathy (77).

While trauma itself degrades the glycocalyx, the choice of solution for intravenous resuscitation also contributes. When healthy subjects were administered 0.9% saline, Hartmann’s solution, 4% and 20% albumin in a double-blind crossover study, only the 0.9% saline produced evidence of glycocalyx degradation through increased plasma syndecan-1 (78). Though the fluid treatment was relatively mild in healthy subjects, it suggested that 0.9% saline, the most widely used resuscitation fluid globally, may be the harshest on the endothelial glycocalyx integrity. Indeed, normal saline was associated with poorer outcomes as compared to other crystalloids in critically ill adults (79), though glycocalyx integrity was not specifically investigated. Additionally, an *in vitro* study suggests hypernatremia, can be associated with normal saline infusion, damaging the endothelial glycocalyx, releasing HA and syndecan-1 (80).

Hemorrhagic shock patients showed significantly increased plasma syndecan-1 concentrations after their injury compared to healthy controls (81). After resuscitation with fresh frozen plasma, syndecan-1 significantly decreased, but it was still elevated over healthy controls (81). This latter response could be due, at least in part, to dilution by the administered fresh frozen plasma; however, *in vitro* studies suggest that fresh frozen plasma reduces endothelial permeability and aids syndecan-1 restoration (81). Indeed, administration of fresh frozen plasma to non-bleeding critically ill patients (45% sepsis patients) resulted in a significantly lower syndecan-1 plasma concentration when compared to plasma levels obtained before transfusion (565 vs. 675 pg/mL) (82). As cytokine/chemokine plasma concentrations were unaffected, and ADAMTS13 concentrations were increased, this latter data suggested that the reduced plasma syndecan-1 concentration was not dilutional. When patients with thoracic aortic dissection received an intravenous product made from pooled, solvent and detergent-treated plasma (OctaplasLG) during surgical repair, their plasma syndecan-1 levels were significantly reduced, indicating less glycocalyx injury (83).

The resuscitation of hemorrhagic trauma patients favors a saline-restricted approach, and emphasizes balanced transfusions that include fresh frozen plasma. While many studies have measured syndecan-1 concentrations after fluid resuscitation, most measured only a single time point after resuscitation and few monitored a time-course. Thus, the positive effects of fresh frozen plasma on the vascular glycocalyx requires further investigation.

### Diabetes

Glycocalyx degradation in diabetes (e.g., hyperglycemia) or diabetes-associated complications [e.g., the systemic inflammatory response associated with diabetic ketoacidosis (84–87)] has not been studied as extensively as compared to other diseases with a severe inflammatory component. Nevertheless, the current knowledge indicates that the endothelial glycocalyx is degraded by acute hyperglycemia and that the endothelial glycocalyx thinning due to hyperglycemia contributes to impaired wound healing in diabetes patients (88).

In a small study of healthy males, acute hyperglycemia induced by a concentrated glucose infusion decreased systemic endothelial glycocalyx thickness, an observation that coincided with a rapid increase in circulating plasma levels of HA (89). This glycocalyx thinning was independent of rapid changes in osmolality, as the glycocalyx did not degrade in response to mannitol. Importantly, the glycocalyx thinning was ameliorated by co-infusion of the antioxidant N-acetyl cysteine, suggesting an important role for ROS. In another study, baseline systemic glycocalyx thickness was significantly decreased in type-1 diabetes patients, compared to controls; and there was a further decrease in type-1 diabetes patients with microalbuminuria (90). The measure of systemic glycocalyx thickness significantly correlated (*r* = 0.73, *p* < 0.01) with sublingual glycocalyx thickness measurements (90). Plasma HA concentrations were also increased in type 1 diabetes patients compared to controls, and again microalbuminuria increased this further (90). In contrast, another trial of 136 type-1 diabetes patients failed to show an association between glycocalyx thickness in sublingual micro-vessels and the level of albuminuria, although the diabetes patients did have a significantly decreased glycocalyx compared to controls (91).

Similar to type-1 diabetes, patients with type-2 diabetes showed a thinner baseline glycocalyx in sublingual and retinal vessels compared to normal controls; additionally these patients had increased plasma HA and hyaluronidase concentrations (92). First degree relatives of type-2 diabetes patients that are insulin resistant, and subjects with dysglycemia showed a lower baseline glycocalyx thickness in sublingual microvessels compared to normal controls (93). Furthermore, the glycocalyx acutely thinned in these subjects during oral glucose tolerance tests, while the thinning was not observed in healthy subjects (93).

While most glycocalyx studies in diabetes have focused on HA, plasma syndecan-1 levels are also significantly increased in type-2 diabetes patients compared to controls (94). Of the diabetes patients, mean fasting blood glucose was 10.32 mM and 29.3% had glucose in their urine. Another study of type-1 diabetes patients found significantly increased plasma concentrations of syndecan-1 in those with nephropathy compared to type-1 patients without (95). Again, the nephropathy patients’ mean glycated hemoglobin was 9.18% vs. 7.58% for controls (normal 4–5.6%), suggesting the nephropathy patients blood glucose was not well controlled. Contrary to this, blood syndecan-1 levels in type-1 diabetic patients with microalbuminuria were increased as compared to patients without, and healthy controls, but no difference in glycated hemoglobin was found (96).

## Strategies/Therapies for Protecting/Restoring Glycocalyx

Because of the extended time-course of glycocalyx recovery (97), and in light of the apparently long-lasting glycocalyx effects of COVID-19 (65, 66), therapies that protect the endothelial glycocalyx or support its replacement would be advantageous. Currently, there is limited data on trials or therapies in humans, likely due to the relatively brief time since the endothelial glycocalyx has gained prominence and the long timelines for clinical research. Many excellent basic science and pre-clinical models have emerged and have recently been reviewed elsewhere (98, 99) so here we will focus primarily on therapies which have human data.

### Steroids

Glucocorticoids have a long history as anti-inflammatory agents; decreasing pro-inflammatory molecule release and reducing extravasation of white blood cells, making steroids an attractive agent for indirectly protecting the glycocalyx. A small trial showed pre-surgical administration of hydrocortisone to patients undergoing cardiac surgery with cardiopulmonary bypass significantly decreased plasma concentrations of heparan sulfate, but not syndecan-1, when compared post-operatively to untreated controls (100). Recently, a phase 2 study investigated the preoperative administration of dexamethasone on abdominal surgery patients with or without albumin, and found that there was no difference in post-operative day 1 plasma syndecan-1. However, the dexamethasone plus albumin group did have lower plasma heparan sulfate (101) concentrations. While this was a small trial with a total of 72 patients, and the improved glycocalyx markers were not substantial, the experimental treatment was well tolerated and there were fewer complications in the experimental group suggesting that the results may improve with different formulations. A single pre-operative dose of methylprednisolone was shown to cause a modest, but significant reduction in post-operative syndecan-1 concentrations compared to controls in patients undergoing total knee arthroplasty (102). As the studies examining the effects of steroids on the glycocalyx have focused on surgical outcomes, it is unclear if they would be effective in critical illness and prolonged inflammation. Critically ill COVID-19 patients respond well to steroid therapy, and glycocalyx degradation is a key pathophysiological mechanism in these patients, raising the possibility that the glycocalyx may be a potential steroid target (103).

### Protease Inhibitors

A variety of proteases cleave syndecans and CD44 (see Table 1), making proteases an attractive target for reducing glycocalyx degradation and/or aiding the glycocalyx recovery. Tranexamic acid is a synthetic lysine derivative that inhibits plasminogen activation and its binding to fibrin, thereby inhibiting fibrinolysis (104). A recent study showed pre- and peri-operative administration of tranexamic acid in patients undergoing posterior lumbar fusion surgery significantly inhibited the 2 h postoperative increase in plasma syndecan-1 compared to untreated patients (105). A study of patients with moderate to severe traumatic brain injury showed a modest but significant decrease in plasma syndean-1 when tranexamic acid was administered within 2 h postinjury (106). While the exact mechanism of syndecan-1 preservation is unknown, it has been suggested from *in vivo* experiments that tranexamic acid additionally inhibits MMP activity (107) as well as fibrinolysis. Tranexamic acid’s effect on the glycocalyx in critical illness has not been thoroughly studied, and although its effect limiting syndecan-1 shedding is promising, its action of inhibiting fibrinolysis (104) suggests it may be incompatible with conditions exhibiting DIC where it may stabilize pathological clots.

The relatively large number of MMPs which can cleave syndecans-1 and 4, and CD44 (Table 1) make MMPs an important target to rescue the glycocalyx. Despite musculoskeletal syndrome as a side effect of early broad-spectrum MMP inhibitors, several more selective MMP inhibitors, particularly those that inhibit MMP-2 or –9, have shown clinical benefit (108). These latter MMP inhibitors are protein-based, including antibodies and tissue inhibitors of MMPs (TIMPs), several of which are currently undergoing clinical trials (109), including an anti-MMP-9 antibody (110). Small peptide inhibitors of ADAM-17, which cleaves syndecan-1 and 4, have also been identified as potential therapeutics (109).

Due to the hypercoagulation that often accompanies critical illnesses, and because thrombin cleaves syndecan-4 (37) it is a rational a target for treatment. While direct thrombin inhibitors have not been specifically evaluated in trials of glycocalyx degradation, there are several compounds available for human use which may provide therapeutic benefit (111, 112).

Finally, the protease inhibitor Ulinastatin, which inhibits lipopolysaccharide-induced heparanase expression and activity, suppressed vascular permeability and HS degradation in mouse models (98).

### Heparinoids/Glycosaminoglycans

Prophylactic administration of low molecular weight heparin lowered heparanase activity in non-ICU COVID-19 patients, though no differences in plasma HS were found, and it did not affect heparanase activity in ventilated COVID-19 patients (62). Other glycocalyx markers were not measured. Heparin can act to protect the glycocalyx *in vitro* and even help reconstitute it by mobilizing syndecan-1 (113).

Endogenous plasma hyaluronidase inhibitors increased during sepsis (18), suggesting exogenous inhibitor addition may be of benefit. The heparinoid Sulodexide is a heterogeneous mixture of sulfated glycosaminoglycans and acts as a heparinase inhibitor. Two months of Sulodexide treatment, increased sublingual, and retinal glycocalyx thickness in type-2 diabetes patients without increased plasma HA concentrations (92). A recent meta-analysis concluded that Sulodexide was reno-protective and decreased albumin excretion rate by 50% in patients with diabetic nephropathy (114). Since glycocalyx degradation in kidney endothelial cells was suspected to play a role in the pathogenesis of diabetic nephropathy (115), this latter finding suggested that Sulodexide may have restored the glycocalyx. Additionally, this data suggested that hepariniod therapy may be useful in the context of the long-term damage associated with COVID-19 (66, 67). Currently, heparin is often used as an anticoagulant in sepsis and COVID-19 patients; however, we are unaware of studies that have measured its effect on the glycocalyx in these diseases.

### Resuscitation Strategies

Volume resuscitation with plasma or plasma products has recently gained attention. As discussed above (*Trauma—Fluid resuscitation*), administering fresh frozen plasma to non-hemorrhagic critically ill patients protected the glycocalyx (82); although plasma treatment for sepsis has been generally not recommended (116). Fluid resuscitation of septic shock patients with albumin-supplemented crystalloids did not affect plasma syndecan-1 concentrations, although there was some possible endothelial protections (46). Moreover, a rodent hemorrhage model suggested albumin administration may partially restore the glycocalyx (117) and a pilot study in human septic shock patients indicated that an albumin infusion may restore endothelial function (118).

Sphingosine 1-phosphate has been shown to correlate with positive outcomes in septic shock (46), and the glycocalyx protective effects of plasma or its products may be a result of the sphingosine 1-phospate present in those products (99).

Although two recent clinical trials demonstrated early pre-hospital plasma administration to trauma patients conferred a significant survival benefit (119), there was no analysis of endothelial glycocalyx markers. Fresh frozen plasma was shown to help restore endothelial syndecan-1 *in vitro* (81), and another study showed early plasma resuscitation can restore the glycocalyx, as opposed to late plasma treatment that was ineffective (120). As discussed above, glycocalyx degradation was reduced by administration of OctaplasLG, as compared to fresh frozen plasma in patients undergoing emergency surgery for thoracic aorta dissection (83). Rodent studies of hemorrhagic shock indicated that plasma resuscitation (either fresh or fresh-frozen) protected or restored the endothelial glycocalyx, where lactated Ringer’s or hydroxyethyl starch solution did not, and increased survival may have been dependent on syndecan-1 restoration [Reviewed in (121)].

### Anti-inflammatory Therapies

Prophylactic administration of a TNF-α inhibitor ameliorated glycocalyx degradation in response to low-dose endotoxin in healthy volunteers (12), and a combination therapy of methotrexate and/or a TNF-α inhibitor in rheumatoid arthritis patients decreased syndecan-1 for greater than 6 weeks (122). However, whether TNF-α inhibition would be beneficial to the glycocalyx in the often more severe inflammation observed in critically ill patients is undetermined, although the history of TNF-α inhibition trials (123, 124) in sepsis would suggest not. Activation of the Tie2 receptor *in vitro* may promote recovery of the endothelial glycocalyx (125); in fact, the Tie2 agonist AV-001 is currently in a phase 2a trial for patients with severe COVID-19 (clinicaltrials.gov ID NCT05123755).

## Concluding Remarks

Several major glycocalyx components have been used as biomarkers to distinguish sub-populations in critical illness or predict outcomes. Glycocalyx degradation occurs during critical illness/injury, but it is not the only pro-inflammatory and pro-thrombotic change to take place, and it must be viewed in context with other microvascular pathologies, endothelial dysfunction, leukocyte/platelet adhesion, and the release of inflammatory mediators, such as cytokines, proteases and ROS. Furthermore, glycocalyx degradation may not be a general response to inflammation; in fact, in some pathologies it may offer disease specific consequences (i.e., trauma-induced auto-heparinization vs. sepsis-induced DIC). Despite an abundance of basic and pre-clinical research into glycocalyx preserving and rebuilding strategies, there remains a lack of clinical trials in humans.

## Author Contributions

EP: data collection, data interpretation, and manuscript writing. GC and DF: concept, data interpretation, critical review of the manuscript, and submission. All authors contributed to the article and approved the submitted version.

## Conflict of Interest

The authors declare that the research was conducted in the absence of any commercial or financial relationships that could be construed as a potential conflict of interest.

## Publisher’s Note

All claims expressed in this article are solely those of the authors and do not necessarily represent those of their affiliated organizations, or those of the publisher, the editors and the reviewers. Any product that may be evaluated in this article, or claim that may be made by its manufacturer, is not guaranteed or endorsed by the publisher.
